# Optimization of Heat–Moisture Treatment Conditions for High-Amylose Starch and Its Application in High-Resistant Starch Triticale Noodles

**DOI:** 10.3390/foods13172724

**Published:** 2024-08-28

**Authors:** Hua Li, Hua Li, Yu Liu, Ruixin Liu, Sirithon Siriamornpun

**Affiliations:** 1 Department of Cuisine and Nutrition, Yangzhou University, Yangzhou 225127, China; 2 Key Laboratory of Chinese Cuisine Intangible Cultural Heritage Technology Inheritance, Ministry of Culture and Tourism, Yangzhou 225127, China; 3 Research Unit of Thai Food Innovation (TFI), Mahasarakham University, Kantarawichai 44150, Thailand; 4 Department of Food Technology and Nutrition, Faculty of Technology, Mahasarakham University, Kantarawichai 44150, Thailand

**Keywords:** starch digestibility, resistant starch, starch structure, heating time, relative crystallinity, pasting property, infrared spectroscopy

## Abstract

Heat–moisture treatment (HMT) is a widely used method for modifying starch properties with the potential to reduce the digestibility of high-amylose starch (HAS). This study aimed to optimize the HMT conditions for HAS and apply the resulting HMT-HAS to triticale noodles to develop low-glycemic-index products. HMT significantly increased the resistant starch (RS) content and decreased the rapidly digestible starch (RDS) content of HAS. The treatment conditions—temperature, heating time, and moisture content—were found to significantly influence the starch composition. Optimal HMT conditions were determined using response surface methodology: a temperature of 108 °C, a heating time of 5.8 h, and a moisture content of 25.50%. Under these conditions, the RS content of HMT-HAS was 60.23%, nearly double that of the untreated sample. Increasing the level of HMT-HAS in triticale noodles led to significant decreases in short-range order, relative crystallinity, and viscosities, while the RS content increased from 12.08% to 34.41%. These findings suggest that incorporating HMT-HAS into triticale noodles effectively enhances starch digestive resistance, supporting the development of functional, low-glycemic-index triticale-based foods.

## 1. Introduction

High-amylose maize starch, abbreviated as high-amylose starch (HAS), could decrease blood glucose levels, increase satiety, improve the gut microbiota, and reduce the risk of obesity and cardiovascular disease [1]. HAS contains a much higher content of resistant starch (RS) than low- or normal-amylose starch, which makes it digested and absorbed by the body at a slower rate, thereby lowering the glycemic index (GI) of food and improving the postprandial glucose and insulin responses [2]. Therefore, HAS has been considered a good raw material for the development of low-GI foods.

Heat–moisture treatment (HMT) is a physical modification technique that involves treating starch at high temperatures (90–130 °C) with low moisture content (15–35%) for a certain period. During HMT, the movement of water molecules is intensified by thermal energy to modify the internal structure of starch [3]. HMT may affect the sensitivity of digested enzymes to starch, depending on the source and variety of the starch as well as the treatment conditions [4]. For example, starch, particularly HAS, is apt to be converted into forms with low GI by HMT [5]. Animal studies have indicated that HMT-HAS exhibits stronger resistance to enzyme digestion in the mouse digestive tract [6] and could effectively reduce plasma cholesterol levels in ovariectomized rats [7]. Therefore, optimizing the HMT conditions for HAS is essential for regulating the GI, promoting the application of HMT technology, and enhancing the utilization of HAS.

Noodles are a popular staple in the world, especially in China, and are generally regarded as a high-GI food. To the best of our knowledge, we are the first to report on the development of low-GI triticale noodles as an alternative product. Triticale (× *Triticosecale* Wittmack), a hybrid grain from wheat (*Triticum* sp.) and rye (*Secale cereale* L.), has been increasingly incorporated into various foods such as bread, biscuits, tortillas, pasta, and noodles due to its high nutritional value and health-promoting properties [8]. Previous research, such as Lorenz et al. [9], has demonstrated the suitability of triticale for noodle production based on its cooking and sensory properties. Additionally, studies have explored the nutrients, polyphenols, and sensory qualities of triticale noodles [10,11], with Liu et al. [12] advancing the development of the gluten network in triticale whole-wheat noodles using enzymes like glucose oxidase, xylanase, and laccase. Compared to traditional wheat noodles, triticale noodles offer superior nutritional value and bioactivity, attributed to higher contents of protein, minerals, vitamins, and phenolics [13]. Despite the growing interest in low-GI foods for managing postprandial blood glucose, limited research has focused on reducing the GI of triticale noodles. Although numerous studies have investigated the effects and mechanisms of HMT on the physicochemical properties and starch digestibility of various starches, the results have been inconsistent. Therefore, optimizing HMT conditions for HAS, and considering factors like heating time, temperature, and moisture content, remains crucial to enhancing the RS content.

Therefore, in this study, we optimized the HMT conditions for HAS by response surface methodology using RS content as the response variable and then incorporated the HAS produced by HMT at optimal conditions into the triticale noodle preparation to attenuate the starch digestibility of the noodle. Meanwhile, some physical characteristics, including microstructure, crystalline characteristics, pasting properties, and short-range order, were also explored to better understand the change in the noodle’s starch characteristics because of the addition of HMT-HAS. This investigation may be helpful in developing low-GI triticale products to meet the needs of diabetes patients and increase the added value of triticale as well.

## 2. Materials and Methods

### 2.1. Materials and Reagents

The triticale cultivar (ND2201) was obtained from Huinong Fumin Technology Co., Ltd. (Beijing, China). High-amylose maize starch (1945, 71% amylose) was purchased from Kehe Biotechnology Co., Ltd. (Shanghai, China). Amyloglucosidase and α-amylase were purchased from Aladdin Corp. (Shanghai, China). Phenol and 3,5-dinitrosalicylic acid were acquired from Suyi Chemical Reagent Co., Ltd. (Shanghai, China) and Lanji Technology Development Co., Ltd. (Shanghai, China), respectively. All other reagents of analytical grade were supplied by Sinopharm Chemical Reagent Co., Ltd. (Shanghai, China).

### 2.2. HMT of HAS

HAS was heat–moisture treated as follows: The moisture content of HAS in a reaction vessel was adjusted to 15–35% by adding distilled water, followed by standing at room temperature for 24 h, and then the sealed vessel was heated in a drying oven (DHG-9055A, Yiheng Technology, Shanghai, China) at a certain temperature (90–130 °C) for a certain period of time (2–6 h). Subsequently, the vessel was cooled for 30 min, and then the samples treated with heat and moisture were dried at 45 °C for 12 h. Finally, the dried samples were ground (Q-250A, Bingdu Electrical Appliance, Shanghai, China) and passed through an 80-mesh sieve to obtain the HMT-HAS samples.

### 2.3. Optimization of HMT Conditions for HAS

#### 2.3.1. Single-Factor Experimental Design

Three independent variables, i.e., moisture content, treatment temperature, and heating time, were investigated for their effects on the starch digestibility of HAS. The experiments were designed as follows: (1) The temperature and time were 110 °C and 4 h, respectively; the moisture content was 15%, 20%, 25%, 30%, or 35%. (2) The moisture content and time were 25% and 4 h, respectively; the temperature was 90 °C, 100 °C, 110 °C, 120 °C, or 130 °C. (3) The moisture content and temperature were 25% and 110 °C, respectively; the time was 2, 3, 4, 5, or 6 h.

#### 2.3.2. Response Surface Experimental Design

To obtain the optimal HMT conditions for HAS, on the basis of the preliminary single-factor experiments, moisture content, treatment temperature, and heating time were set as the independent variables, with RS content as the response variable. The Box–Behnken design was conducted using Design Expert 13 (Stat-Ease, Minneapolis, MN, USA). The factors and levels of the response surface experiment are shown in Table 1.

### 2.4. Preparation of Triticale Noodle Samples

The triticale noodles were prepared as follows: Briefly, 100 g of the flour (consisting of triticale and HMT-HAS) containing 0%, 10%, 20%, 30%, 40%, or 50% HMT-HAS was blended with 60 mL of water and kneaded into a uniform dough. Subsequently, the dough was set to rest for 30 min at 30 °C, followed by being cut into noodles that were 1 mm thick, 5 mm wide, and 20 cm long. After being freeze-dried, some of the noodles were ground into a powder, passing through an 80-mesh sieve. Both the ground and unground noodles were sealed and preserved in a desiccator at room temperature until used.

### 2.5. Scanning Electron Microscopy Analysis

As per the method supplied by Lv [14], the microstructural characteristics of freeze-dried noodle samples were explored using a scanning electron microscope (S-4800II, Hitachi, Tokyo, Japan) with a magnification of 800× at an accelerating voltage of 20 kV.

### 2.6. Analysis of Short-Range Order

The short-range order of the freeze-dried noodle powders was investigated using Fourier transform infrared spectroscopy (670-IR+610-IR, Varian, Palo Alto, CA, USA), as reported by Zhang [15]. The scan was performed in the range of 400–4000 cm^−1^ with a resolution of 4 cm^−1^. The spectra were obtained by scanning 32 times with KBr as the background and analyzed using OMNIC 9.7 (Thermo Fisher Scientific Inc., Waltham, MA USA). The spectra were deconvoluted and normalized in the range of 900 to 1250 cm^−1^, with a peak width of 40 cm^−1^ and an enhancement factor of 1.9.

### 2.7. X-ray Diffraction Analysis

Following the method of Li et al. [16], the X-ray diffraction patterns of the freeze-dried noodle powders were determined using an X-ray diffractometer (D8 Advance, Bruker AXS, Karlsruhe, Baden-Württemberg, Germany) at 40 kV and 40 mA with Cu-Kα radiation. The scanning region ranged from 5° to 40° (2*θ*) at a scanning speed of 2°/min. The relative crystallinity was calculated using the X-ray diffractometer software (Topas 4.0).

### 2.8. Determination of Pasting Properties

The pasting properties of the freeze-dried noodle powders were analyzed with a rapid viscosity analyzer (TechMaster RVA, Perten, Segeltorp, Sweden). A mixture of 1.5 g of the noodle sample and 25 g of distilled water was put into an aluminum box for RVA. The program parameters were set as follows: equilibration at 50 °C for 1 min, heating to 95 °C at a rate of 12 °C/min, holding at 95 °C for 2.5 min, and then cooling to 50 °C at a rate of 12 °C/min and holding for 2 min. The rotational speed of the helical rotor was 960 rpm for the first 10 s and then reduced to 160 rpm.

### 2.9. Analysis of Total Starch Content

The total starch content was analyzed using the acid hydrolysis method outlined in the “National Food Safety Standard—Determination of Starch in Foods (GB5009.9-2016)” [17].

### 2.10. Analysis of In Vitro Starch Digestibility

The RDS, SDS, and RS contents were analyzed following the method reported by Englyst et al. [18] with minor modifications. In brief, 200 mg of the sample was thoroughly mixed with 15 mL of sodium acetate buffer (0.2 mol/L, pH 5.2) and then incubated in a boiling water bath for 15 min. Following a water bath at 37 °C for 5 min, the sample was hydrolyzed with 5 mL of α-amylase (290 U/mL) and 5 mL of amyloglucosidase (15 U/mL) at 160 rpm and 37 °C. After hydrolysis for 20 and 120 min, 1 mL of the hydrolysate was immediately blended with 4 mL of a 66% anhydrous ethanol solution. Following centrifugation (Allegra X-30R, Beckman Coulter, Brea, CA, USA) at 6000 rpm for 10 min, the level of glucose in the supernatant was measured using the DNS method.

### 2.11. Statistical Analysis

The data were presented as the mean ± standard deviation for three replications. Significance analysis was carried out using SPSS 19.0 (IBM, Armonk, NY, USA) at the *p* < 0.05 level. Graphs were constructed by Origin 2018 (OriginLab, Northampton, MA, USA).

## 3. Results and Discussion

### 3.1. Single Factor Analysis for HMT

#### 3.1.1. Effect of Moisture Content

Starch digestibility has been demonstrated to be dependent on the parameters in HMT, including moisture content, treatment temperature, and heating time [19]. Table 2 shows the effect of moisture content during HMT on the starch composition of HAS. HMT led to an evident change in the contents of RDS, SDS, and RS. Moreover, the contents of those different types of starches varied significantly with the moisture content. Compared to the untreated counterpart, the RDS content decreased by 21–50% and the RS content increased by 31–80%, with the highest RS and lowest RDS contents observed at 25% of moisture content. The impact of HMT on starch digestibility may be attributed to starch structural and morphological changes under high temperature and low moisture content, such as crystallite disruption, crystallite reorientation, amylose–lipid interaction, starch chain interaction, and formation of fissures and cracks on the granule surface [19]. In our study, the reasons for native starch showing the highest RDS content were likely as follows: HMT can cause the formation of ordered molecular aggregation architecture, which increases crystalline perfection and improves resistance to enzymes [20]. In addition, it has been reported that there is up to 1.0% lipid contents in typical washed cereal starch [21]. Therefore, the starch–lipid complex, which does not fit into the active sites of α-amylases, may be formed during HMT, as reported by Liu et al. [22], who also found the formation of the starch–lipid complex after HMT despite the low lipid content (0.5%) in buckwheat starch. At high temperatures, the intensified movement of water molecules disrupts the ordered structure of starch, causing migration and rearrangement. This leads to enhanced interaction between the amorphous regions and the starch molecular chains, thereby obstructing the entry of amylase into the interior of the starch granules and inhibiting starch digestion [23]. When higher moisture levels were used, the disruption of starch structure by HMT might be intensified rather than rearranged [24]. When the moisture content exceeded 25%, with more water molecules at high temperatures, starch gelatinization intensified, leading to structural disruption of starch and increased attachment sites for amylase, thus promoting starch digestion. Therefore, a moisture content of 20% to 30% was selected for the response surface experiment.

#### 3.1.2. Effect of Treatment Temperature

The effect of temperature during HMT on the starch composition of HAS is shown in Table 3. Similarly, the temperature also significantly affected the contents of RDS, SDS, and RS. The sample by HMT at 100 °C exhibited the lowest RDS content, about half of that in the untreated counterpart. Meanwhile, HMT at 100 °C induced an increase in RS content, 1.8 times higher than the untreated one. It may be due to the intensified movement of water molecules under thermal energy, promoting the perfection of crystalline structure and the formation of more stable starch-lipid complexes. When the temperature was higher than 90 °C, the RDS content as well as the RS content at different temperatures was comparable, although significant. It may be attributed to the stable starch structure, in accordance with the findings of Wang et al. [25]. Increasing the temperature leads to a more stable starch structure through the processes of gelatinization and retrogradation. The stability arises from the disruption of the crystalline regions in the starch granules and the formation of a more amorphous, gel-like structure that is less prone to reverting to its original state [26]. Considering the reduction in cost and starch discoloration at excessively high temperatures, temperatures ranging from 90 °C to 110 °C were selected for the response surface experiment.

#### 3.1.3. Effect of Heating Time

Table 4 displays the effect of heating time on the starch composition of the HMT-HAS. The contents of RDS, SDS, and RS varied significantly with time. As the time increased, the RS content greatly increased, reaching a maximum of 62.06% at 6 h, approximately two times that in the untreated sample. On the contrary, the RDS content sharply decreased from 45.76% (the untreated sample) to 19.31% (the sample with HMT for 6 h). This phenomenon may be attributed to the enhanced interaction between the amorphous regions and molecular chains under the influence of water and heat with prolonged HMT time, therefore reinforcing the stability of the internal structure and hindering the entry of digestive enzymes [27]. In the preliminary study, we observed that HMT for more than 6 h could cause starch discoloration and a burnt smell, thereby limiting the treatment time to 4 to 6 h for the response surface experiment.

### 3.2. Response Surface Analysis of HMT Conditions

The HMT conditions were optimized by response surface methodology. The results are shown in Table 5. Through regression analysis, the relationship between the RS content and the independent variables (treatment temperature, heating time, and moisture content) was presented by a quadratic polynomial equation as follows:

RS = 55.67 + 6.05A + 3.56B + 2.20C + 0.58AB + 0.45AC + 0.96BC − 3.33A^2^ − 2.43B^2^ − 3.86C^2^

The variance analysis for the regression equation is displayed in Table 6. The regression model is highly significant (*p* < 0.01), with a non-significant lack of fit, indicating the reasonableness of the regression model and its good fit. Thus, this model could be used to predict the relationship between HMT conditions and RS content. The determination coefficient (*R*^2^) and adjusted coefficient (*R*^2^*_Adj_*) of the equation were 0.9938 and 0.9858, respectively, indicating a high degree of fit between the predicted and actual values. Among these factors, A, B, C, A^2^, B^2^, and C^2^ were highly significant (*p* < 0.01), BC was significant (*p* < 0.05), and the rest were not significant. According to the *F*-values, the influence of HMT conditions on the RS content of HAS was treatment temperature > heating time > moisture content.

Based on the response surface analysis, the optimal HMT conditions for the highest RS content in HAS were as follows: treatment temperature of 108.25 °C, heating time of 5.82 h, and moisture content of 25.54%, with a predicted RS content of 60.43%. Considering operability, the treatment conditions were adjusted to a temperature of 108 °C, a time of 5.80 h, and a moisture content of 25.50%. Validation experiments in triplicate were conducted on the optimal treatment conditions, and the average RS content was 60.23%, close to the predicted value, indicating the reliability and practicality of the model.

### 3.3. Macroscopic and Microscopic Structures

**Figure 1:**
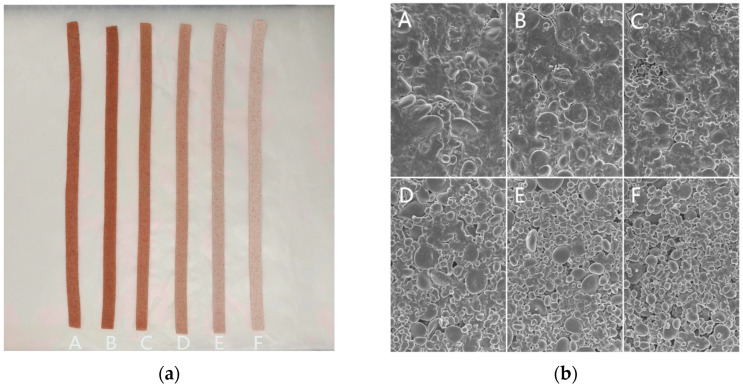
Photographs of triticale noodles with different levels of HMT-HAS: (**a**) macro images; (**b**) SEM micrographs. The levels of HMT-HAS are 0% (**A**), 10% (**B**), 20% (**C**), 30% (**D**), 40% (**E**), and 50% (**F**).

The macroscopic and microscopic structural images of triticale noodles with different levels of HMT-HAS are depicted in Figure 1. At the macroscopic level, pure triticale noodles, due to the presence of bran, exhibited a relatively rough surface, a dark appearance, and a brownish color. The addition of HMT-HAS lightened the color of the noodles, gradually transitioning towards white, and the surface became smooth and elastic, making it more readily accepted sensorially. At the microscopic level, the addition of HMT-HAS led to a smoother surface for the noodles. However, as the protein content decreases, gluten networks may have difficulty forming, and the gel structure gradually becomes loose, even showing tiny pores, in agreement with the findings by Zhang et al. [28]. This microscopic change inevitably affects the texture and mouthfeel of the noodles.

### 3.4. Short-Range Order

**Figure 2:**
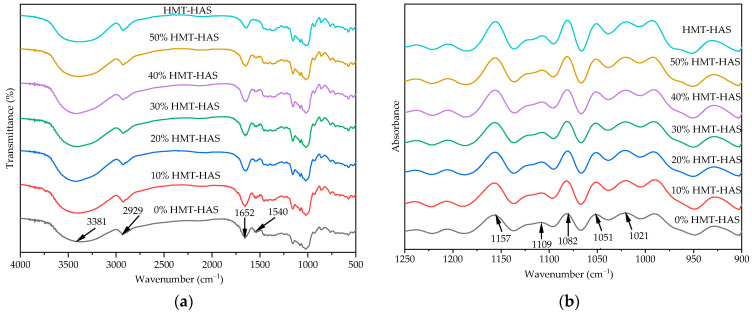
Fourier transform infrared spectra of triticale noodles with different levels of HMT-HAS: (**a**) transmittance spectra; (**b**) deconvoluted spectra.

The Fourier transform infrared spectra of the triticale noodles with different levels of HMT-HAS are illustrated in Figure 2, revealing similar peak distributions among the spectra with varying addition levels and HMT-HAS. The broad band at 3381 cm^−1^ was related to the stretching vibration of O-H, while the characteristic peaks at 2929 and 1652 cm^−1^ corresponded to the C-H deformation vibration of the glucose element and the bending vibration of O-H in water, respectively, which is associated with the amorphous region of starch [29]. The peaks at 1157, 1109, and 1082 cm^−1^ were attributed to the asymmetric C-O and C-C skeleton stretching vibrations [29,30]. However, the triticale noodle samples showed a peak at 1540 cm^−1^, corresponding to the stretching vibration of protein amide II [31], which did not exist in HMT-HAS. The IR bands at ca. 1047 and ca. 1022 cm^−1^ are sensitive to the amount of ordered and amorphous structures of starch, respectively, so the absorbance ratio of the two bands could be used to evaluate the short-range order of double helices [29]. Our preliminary data have shown that the short-range order of HMT-HAS (0.860) was significantly lower than that of triticale flour (0.964). Therefore, we tried to determine how the short-range order changes when adding HMT-HAS to the triticale noodle. As shown in Table 7, with the increasing level of HMT-HAS, the absorbance ratio at R_1051 cm^−1^_ and R_1021 cm^−1^_ decreased from 0.936 to 0.883, suggesting a notable weakening of the short-range order of starch in the noodles. In other words, there was an inverse relationship between the amylose content and the content of the short-range ordered structure, consistent with the findings of Shi et al. [32] and Karwasra et al. [33]. Starch with a high content of amylose tends to have relatively low short-range order, possibly because amylose interferes with the formation of double-helix structures between amylopectin molecules.

### 3.5. X-ray Diffraction and Relative Crystallinity

**Figure 3:**
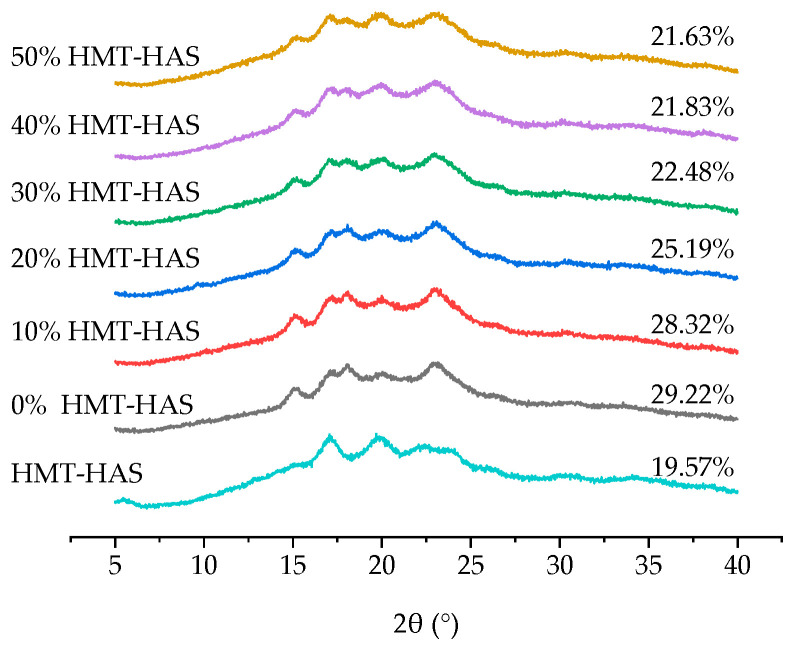
X-ray diffraction patterns of triticale noodles with different levels of HMT-HAS.

The X-ray diffraction patterns and relative crystallinity of the triticale noodles with different levels of HMT-HAS are displayed in Figure 3. The crystalline structure of starch generally includes three main types (A-, B-, and C-type) on the basis of their characteristics and X-ray diffraction patterns [34]. Triticale starch exhibited a typical A-type crystalline structure, with diffraction peaks at 15°, 17°, 18°, and 23° (2θ), whereas HMT-HAS showed single peaks at 5.6°, 15°, and 17° and a merging of doublet at 22° and 24°, still exhibiting a B-type structure, although partly changed by HMT. Additionally, HMT-HAS showed a peak at 20°, indicating the presence of a V-type crystalline structure. Undoubtedly, after the addition of HMT-HAS, a prominent V-type diffraction peak appeared at 20°. Moreover, with the level of HMT-HAS increasing, the peak became more pronounced, indicating an increase in the content of amylose–lipid complexes [35]. Due to the increasing level of HMT-HAS, the content of amylose in the noodles increased, and the amorphous region expanded. Consequently, the relative crystallinity decreased from 29.22% to 21.63%.

### 3.6. Pasting Properties

The pasting properties of a food reflect the changes in viscosity during the heating-cooling process in the presence of water, and viscosity development is closely related to the formation of a tightly packed array of swollen and deformable granules and the leaching of amylose [36]. These changes, mainly induced by the alteration of starch structure, may affect the sensory characteristics and starch digestibility of the food product.

As shown in Table 8, the addition of HMT-HAS significantly reduced the peak viscosity, trough viscosity, final viscosity, breakdown, setback, and peak time of triticale noodles. The pasting properties of starch were influenced by some factors such as amylose content and starch molecular structure [37]. The addition of HMT-HAS led to an increase in the ratio of amylose to amylopectin in triticale noodles. Amylopectin can enhance the cohesion and network structure of starch granules, thus increasing viscosity during starch gelatinization. Therefore, there was a significant negative correlation between these indicators of viscosity and the amylose/amylopectin ratio [38]. The phenomenon was also observed in wheat starch: as the amylose content increased, these values for pasting properties—peak and final viscosities and breakdown—all had a tendency to decrease [39]. This implied that with the increase in amylose content, the viscosity characteristics of starch showed a gradual weakening trend. Additionally, HMT resulted in a stable internal structure of starch, increased heat stability, and a low water-binding capacity, thus reducing viscosity and decreasing setback value [40].

The pasting properties of starch are closely related to noodle quality. Hormdok and Noomhorm [36] investigated the effects of HMT-rice starch substitution on the cooking and textural properties of rice noodles and found that cooking loss correlated positively with breakdown, while tensile strength and extensibility of the noodles correlated negatively with setback. Whereas, for Chinese dry white noodles, peak viscosity and breakdown were positively associated with the elasticity, stickiness, and smoothness of the noodles [41]. Similarly, for Chinese fresh white noodles, breakdown exhibited a significant positive correlation with the noodles’ viscoelasticity and smoothness [42]. Consequently, the decrease in sensory quality of triticale noodles probably occurs at high levels of HMT-HAS; thus, further studies for improving that aspect are needed.

### 3.7. In Vitro Starch Digestibility

As shown in Table 9, the addition of HMT-HAS caused a significant reduction in the contents of RDS and SDS in the noodles, while the RS content increased. Punia [43] believed that the content of amylose may be the main factor influencing almost all the physicochemical properties of barley starch. Similarly, starch digestibility was also influenced by amylose content, and the RS content of starch usually increased with an increase in amylose content [44]. In addition, through optimal HMT conditions, the RS content of HAS increased from 30.98% to 60.23%, so the higher the amount added to the noodles, the higher the RS content. When the addition level reached 50%, the RS content of the noodles was 34.41%, nearly three times higher than that of pure triticale noodles. Similarly, Li et al. [45] reported that the RS content significantly rose by about 6 times when 50% high-amylose wheat flour was added to wheat bread. The results proved that the addition of HMT-HAS could significantly decrease the starch digestibility of triticale noodles, which helps in the development of functional triticale noodles.

GI is a commonly used metric for estimating the postprandial blood glucose response to carbohydrate-containing foods. It is well established that diets low in GI are associated with the prevention and management of diabetes and coronary heart disease [46]. The FAO recommended increasing the intake of low-GI foods, particularly for individuals with diabetes and impaired glucose tolerance [47].

While it is recognized that some inconsistencies exist between GI values predicted by in vitro digestion models and those obtained from human studies, in vitro models are generally considered reliable estimates of GI for foods [48,49]. RS is a type of starch that resists digestion in the gastrointestinal tract and is instead fermented by microorganisms in the colon. There is a strong negative correlation between RS content and GI value, making RS a useful proxy for estimating the potential GI of food products [50].

In our study, we utilized the RS content to infer the starch digestibility of triticale noodles. However, we acknowledge that this is an indirect measure, and the actual GI value of the final triticale noodle product should be validated through human trials to confirm our findings.

## 4. Conclusions

To promote the application of HAS in low-GI triticale noodles, we investigated the effects of HMT parameters on the starch digestibility of HAS by response surface methodology. As expected, the RS content was significantly enhanced, but the RDS content was reduced after HMT. The optimal HMT conditions for improving the RS content of HAS were a temperature of 108 °C, a time of 5.8 h, and a moisture content of 25.50%. Under these conditions, the RS content reached up to 60.23%, approximately 1.94 times higher than the untreated counterpart. The results indicated that HMT may be an effective means to attenuate starch digestibility for the development of low-GI foods. The addition of HMT-HAS to the triticale flour significantly affected the physicochemical properties of triticale noodles. With the level of HMT-HAS increasing, the triticale noodles showed a lighter color and a smoother surface, but the gluten network may be weakened. Additionally, the short-range order, relative crystallinity, pasting properties, and starch digestibility of the noodles all decreased. Particularly when the level of HMT-HAS reached 50%, the RS content of triticale noodles greatly increased to 34.41%, approximately three times higher than that of the pure triticale noodles, significantly enhancing the resistance to digestion of triticale noodles. According to the literature and current information, there are no official recommendations for RS intake. Numerous studies suggest that different amounts of RS can positively impact health [51]. Some researchers recommend a beneficial intake of RS to be at least 14% of total starch, or around 20 g per day, or 6 g per meal. It has been shown that consuming 6–12 g of RS per meal can enhance postprandial glucose and insulin responses. However, for additional health benefits such as increased stool volume, a daily intake of approximately 15–20 g is necessary. Therefore, while we cannot provide a precise value or range for the quantity of RS and the level of starch digestibility, we recommend an approximate range (40–80 g/meal) for the consumption of the triticale noodles.

After incorporating 50% HMT-HAS into triticale noodles, the key characteristics of traditional wheat noodles, such as texture, appearance, and processability, were still retained. However, a slight decrease in sensory quality was observed. Further studies for improving that aspect are needed. Based on these findings, we conclude that a 50% HMT-HAS triticale noodle maintains the fundamental qualities of a noodle and can be considered a new variety of noodle. Finally, the principles and methodologies used in this study could be applied to other cereal-based products beyond noodles, such as bread, pasta, and breakfast cereals, to enhance their nutritional profiles and lower their glycemic indices.

## Figures and Tables

**Table 1:** Factors and levels of response surface experiment.

Levels	Factors		
	A: Moisture Content (%)	B: Treatment Temperature (°C)	C: Heating Time (h)
−1	20	100	4
0	25	110	5
1	30	120	6

**Table 2:** Starch digestibility of HAS under HMT with different moisture contents.

Moisture Content (%)	RDS (%)	SDS (%)	RS (%)
untreated	45.76 ± 0.15 ^a^	23.26 ± 0.61 ^c^	30.98 ± 0.67 ^f^
15	32.47 ± 0.14 ^c^	27.09 ± 0.09 ^a^	40.44 ± 0.14 ^e^
20	30.57 ± 0.08 ^d^	24.55 ± 0.48 ^b^	44.87 ± 0.41 ^c^
25	22.79 ± 0.85 ^f^	21.30 ± 0.72 ^d^	55.91 ± 0.13 ^a^
30	29.58 ± 0.21 ^e^	20.62 ± 0.35 ^d^	49.80 ± 0.47 ^b^
35	36.01 ± 0.69 ^b^	20.78 ± 0.82 ^d^	43.21 ± 0.44 ^d^

Values are presented as mean ± standard deviation (*n* = 3), and values with different lowercases in the same column are considered significantly different (*p* < 0.05). HMT: heat–moisture treatment. HAS: high-amylose starch. RDS: rapidly digestible starch. SDS: slowly digestible starch. RS: resistant starch.

**Table 3:** Starch digestibility of HAS under HMT at different treatment temperatures.

Treatment Temperature (°C)	RDS (%)	SDS (%)	RS (%)
untreated	45.76 ± 0.15 ^a^	23.26 ± 0.61 ^ab^	30.98 ± 0.67 ^d^
90	33.81 ± 0.22 ^b^	23.96 ± 0.07 ^a^	42.23 ± 0.24 ^c^
100	22.36 ± 0.87 ^d^	22.34 ± 0.91 ^b^	55.30 ± 0.12 ^b^
110	23.62 ± 0.15 ^c^	20.73 ± 0.40 ^c^	55.65 ± 0.51 ^ab^
120	23.18 ± 0.97 ^cd^	20.91 ± 0.91 ^c^	55.91 ± 0.13 ^ab^
130	23.02 ± 0.23 ^cd^	20.76 ± 0.10 ^c^	56.22 ± 0.14 ^a^

^a–d^: values with different lowercases in the same column are considered significantly different (*p* < 0.05).

**Table 4:** Starch digestibility of HAS under HMT at different heating times.

Heating Time (h)	RDS (%)	SDS (%)	RS (%)
untreated	45.76 ± 0.15 ^a^	23.26 ± 0.61 ^a^	30.98 ± 0.67 ^e^
2	25.90 ± 0.15 ^b^	19.98 ± 0.85 ^b^	54.12 ± 0.73 ^d^
3	24.60 ± 0.28 ^c^	20.94 ± 0.44 ^b^	54.46 ± 0.26 ^d^
4	21.84 ± 0.20 ^d^	22.25 ± 0.27 ^a^	55.91 ± 0.13 ^c^
5	20.62 ± 0.88 ^e^	20.49 ± 0.78 ^b^	58.89 ± 0.90 ^b^
6	19.31 ± 0.48 ^f^	18.63 ± 0.73 ^c^	62.06 ± 0.25 ^a^

^a–f^: values with different lowercases in the same column are considered significantly different (*p* < 0.05).

**Table 5:** Box–Behnken design arrangement and results of response surface experiment.

Run	A: Treatment Temperature (°C)	B: Heating Time (h)	C: Moisture Content (%)	RS (%)
1	100	4	20	44.15
2	90	5	30	44.14
3	90	5	20	40.94
4	110	4	25	52.21
5	110	5	30	56.91
6	100	5	25	56.37
7	100	5	25	55.45
8	100	6	20	49.92
9	100	5	25	56.39
10	100	5	25	55.76
11	90	6	25	46.44
12	110	5	20	51.91
13	100	6	30	56.51
14	100	4	30	46.92
15	110	6	25	59.93
16	90	4	25	41.03
17	100	5	25	54.36

**Table 6:** Variance analysis for regression equation.

Variation Source	Sum of Squares	Degree of Freedom	Mean Square	*F*-Value	*p*-Value	Significance
Model	588.16	9	65.35	124.35	<0.0001	**
A	292.94	1	292.94	557.40	<0.0001	**
B	101.46	1	101.46	193.06	<0.0001	**
C	38.54	1	38.54	73.34	<0.0001	**
AB	1.33	1	1.33	2.54	0.1551	
AC	0.81	1	0.81	1.54	0.2544	
BC	3.65	1	3.65	6.94	0.0337	*
A^2^	46.74	1	46.74	88.93	<0.0001	**
B^2^	24.90	1	24.90	47.38	0.0002	**
C^2^	62.71	1	62.71	119.32	<0.0001	**
Residual	3.68	7	0.5255			
Lack of Fit	0.90	3	0.2993	0.4305	0.7427	
Pure Error	2.78	4	0.6952			
Total Deviation	591.83	16				
				*R*^2^ = 0.9938	*R*^2^*_Adj_* = 0.9858	*C.V*.% = 1.42

* significant (*p* < 0.05); ** highly significant (*p* < 0.01).

**Table 7:** Absorbance ratios at R_1051 cm^−1^_ and R_1021 cm^−1^_ in Fourier transform infrared spectroscopy.

Samples	R_1051 cm^−1^_/R_1021 cm^−1^_
0% HMT-HAS	0.936 ± 0.001 ^a^
10% HMT-HAS	0.909 ± 0.002 ^b^
20% HMT-HAS	0.894 ± 0.002 ^c^
30% HMT-HAS	0.892 ± 0.000 ^c^
40% HMT-HAS	0.889 ± 0.001 ^c^
50% HMT-HAS	0.883 ± 0.005 ^d^
HMT-HAS	0.860 ± 0.002 ^e^

^a–e^: values with different lowercases in the same column are considered significantly different (*p* < 0.05).

**Table 8:** Pasting properties of triticale noodles with different levels of HMT-HAS.

HMT-HAS (%)	Peak Viscosity (cp)	Trough Viscosity (cp)	Breakdown (cp)	Final Viscosity (cp)	Setback (cp)	Peak Time (min)
0	1245.33 ± 45.79 ^a^	953.33 ± 59.88 ^a^	292.00 ± 14.18 ^a^	1870.67 ± 17.79 ^a^	917.33 ± 75.34 ^a^	6.18 ± 0.20 ^a^
10	909.33 ± 39.32 ^b^	670.00 ± 31.22 ^b^	220.33 ± 15.01 ^b^	1523.33 ± 30.62 ^b^	853.33 ± 13.50 ^a^	5.84 ± 0.04 ^b^
20	612.00 ± 23.00 ^c^	477.00 ± 5.29 ^c^	144.00 ± 7.55 ^c^	1112.67 ± 33.84 ^c^	635.67 ± 29.28 ^b^	5.55 ± 0.12 ^c^
30	419.33 ± 3.51 ^d^	333.67 ± 1.53 ^d^	83.67 ± 2.31 ^d^	781.33 ± 4.62 ^d^	447.67 ± 3.21 ^c^	5.35 ± 0.03 ^cd^
40	236.67 ± 2.08 ^e^	203.00 ± 1.73 ^e^	35.67 ± 1.53 ^e^	449.00 ± 6.24 ^e^	246.00 ± 5.20 ^d^	5.25 ± 0.07 ^de^
50	132.00 ± 7.00 ^f^	129.00 ± 7.94 ^f^	4.33 ± 1.53 ^f^	268.67 ± 21.73 ^f^	139.67 ± 17.62 ^e^	5.10 ± 0.09 ^e^

^a–f^: values with different lowercases in the same column are considered significantly different (*p* < 0.05).

**Table 9:** Starch digestibility of triticale noodles with different levels of HMT-HAS.

HMT-HAS (%)	RDS (%)	SDS (%)	RS (%)
0	64.88 ± 0.10 ^a^	23.03 ± 0.08 ^a^	12.08 ± 0.08 ^f^
10	60.35 ± 0.42 ^b^	21.33 ± 0.35 ^b^	18.32 ± 0.34 ^e^
20	57.12 ± 0.35 ^c^	20.29 ± 0.32 ^c^	22.59 ± 0.26 ^d^
30	53.19 ± 0.07 ^d^	19.76 ± 0.26 ^cd^	27.05 ± 0.22 ^c^
40	49.82 ± 0.35 ^e^	19.34 ± 0.45 ^d^	30.84 ± 0.33 ^b^
50	46.96 ± 0.49 ^f^	18.63 ± 0.38 ^e^	34.41 ± 0.17 ^a^

^a–f^: values with different lowercases in the same column are considered significantly different (*p* < 0.05).

## Data Availability

The data presented in this study are available on request from the corresponding authors. The data are not publicly available due to privacy restrictions.
